# Epithelial insulin receptor expression–prognostic relevance in colorectal cancer

**DOI:** 10.18632/oncotarget.26490

**Published:** 2018-12-25

**Authors:** Steffen M. Heckl, Marie Pellinghaus, Sandra Krüger, Clara Bosselmann, Franziska Wilhelm, Hans-Michael Behrens, Stefan Schreiber, Christoph Röcken

**Affiliations:** ^1^ Department of Internal Medicine I, University Hospital Schleswig-Holstein, Kiel, Germany; ^2^ Department of Pathology, Christian-Albrechts-University, Kiel, Germany

**Keywords:** colorectal cancer, insulin receptor, cancer risk factor, cancer prognosis, cancer therapeutic target

## Abstract

**Background:**

Metabolic reprogramming in cancer encompasses the insulin receptor (IR) as a player of energy homeostasis and proliferation. We aimed to characterize vascular (VIR) and epithelial (EIR) IR expression in CRC and correlate it with clinico-pathological parameters and survival.

**Methods:**

1580 primary CRCs were explored by immunohistochemistry for evaluation of VIR and EIR. Subgroup analyses included *in situ* hybridization for IR isoform A (IR-A) and DNA mismatch repair protein immunohistochemistry. Clinico-pathological and survival parameters were studied.

**Results:**

High VIR was evident in 63.5% of all CRC samples and was associated with T-stage (*P* = 0.005). EIR was present in 72.2% and was associated with lower T-stages (*P* = 0.006) and UICC-stages (*P* < 0.001). EIR negativity was associated with increased metastasis (*P =* 0.028), nodal spread (*P* < 0.001), lymphatic invasion (*P =* 0.008) and a decreased tumor-specific (*P =* 0.011) and overall survival (*P =* 0.007; 95%–C.I.: 44.5–84.1). EIR negativity in UICC-stage II was associated with a significantly worse tumor-specific (*P =* 0.045) and overall (*P =* 0.043) survival. IR-A was expressed in CRC vessels and cells.

**Conclusions:**

We demonstrate VIR to be frequent in CRC and characterize EIR negativity as an important prognostic risk factor. The association between EIR negativity and worse survival in UICC-stage II should be prospectively evaluated for an application in therapeutic algorithms.

## INTRODUCTION

Colorectal cancer (CRC) is the third most common cancer in men and the second most common cancer in women worldwide with diverse etiologies [1]. Most frequently, sporadic CRC arises from colorectal adenomas. It may also develop in patients suffering from inflammatory bowel disease, e.g. ulcerative colitis and Crohn’s disease [2]. Finally, CRC may be linked to hereditary cancer syndromes, such as Lynch-syndrome and familial adenomatous polyposis [3]. Irrespective of the etiology, there is ample evidence that development and progression are associated with metabolic reprogramming of proliferating neoplastic cells [4] leading to increased glucose uptake mediated by GLUT transporters [5]. In addition and less well characterized, metabolic reprogramming also encompasses an altered expression of the insulin receptor (IR). The IR is a cell surface receptor, which is involved in energy homeostasis and proliferation. It is up-regulated in various cancers such as colon, thyroid, lung, breast and ovarian cancer [6].

The IR has two isoforms, which differ in their functional properties: the isoform B (IR-B) conveys the classical metabolic effects of insulin [6]. Isoform A (IR-A) exhibits mitogenic and proliferative effects and is upregulated in cancer [6]. IR-A is stimulated by insulin and IGF-II, whereas IR-B binds insulin preferentially.

Overexpression of the IR has been associated with poor survival in a study involving non-small cell lung cancer [7]. Studies about the influence of IR on survival in breast cancer have led to opposing results with one study associating IR-expression with a favourable [8] and another with a worse outcome [9]. Takahashi et al. associated IR-expression in renal cell carcinoma with a significantly longer overall and disease-free survival [10]. All these studies explored the expression of IR in epithelial tumor cells (EIR). However, IR may also be expressed in other tumor compartments, e.g. tumor-vasculature (VIR). Neoangiogenesis is a hallmark of cancer and may also necessitate increased glucose uptake and metabolism.

In this study we aimed to test the following hypotheses: (1) the IR is expressed by different cellular compartments of CRC; (2) IR-A is the isoform preferentially expressed in CRC; and (3) differential expression of IR is associated with clinico-pathological patient characteristics and patient survival.

## RESULTS

### Study population

Table 1 depicts the clinico-pathological characteristics of our patient population, which included 769 women and 811 men. The median age was 71 years (range 16–95). The median follow-up of our population was 60.6 months, during which 785 out of 1580 patients had died.

**Table 1:** Correlation between clinico-pathological patient characteristics and the expression of insulin receptor in endothelial cells of tumor vessels (VIR) as well as tumor cells (EIR)

		Total		Vascular expression (VIR)				Epithelial expression (EIR)				
		*n* (%)		low (0/1+) *n* (%)		high (2+/3+) *n* (%)		negative *n* (%)			positive *n* (%)	
**Gender**	*n p*-Value_(a)_	1580		1580		0.917		1580		0.092		
Male		811	(51.3)	297	(36.6)	514	(63.4)	241	(29.7)	570		(70.3)
Female		769	(48.7)	279	(36.3)	490	(63.7)	199	(25.9)	570		(74.1)
**Age Group**	*n p*-Value_(a)_	1580		1580		0.374		1580		0.866		
< 71 years		777	(49.2)	292	(37.6)	485	(62.4)	218	(28.1)	559		(71.9)
≥ 71 years		803	(50.8)	284	(35.4)	519	(64.6)	222	(27.6)	581		(72.4)
**Localization**	*n p*-Value_(a)_	1499		1499		**0.024**		1499		0.556		
Left-sided		910	(60.71)	313	(34.4)	597	(65.6)	259	(28.5)	651		(71.5)
Right-sided		589	(39.29)	237	(40.2)	352	(59.8)	159	(27.0)	430		(73.0)
**T-Category**	*n p*-Value_(b)_	1580		1580		0.093		1580		**0.006**		
T1		77	(4.9)	34	(44.2)	43	(55.8)	17	(22.1)	60		(77.9)
T2		329	(20.8)	138	(41.9)	191	(58.1)	82	(24.9)	247		(75.1)
T3		908	(57.5)	302	(33.3)	606	(66.7)	249	(27.4)	659		(72.6)
T4a/b		266	(16.8)	102	(38.3)	164	(61.7)	92	(34.6)	174		(65.4)
**T-Category (grouped)**	*n p*-Value_(a)_	1580		1580		**0.005**		1580		0.072		
T1/T2		406	(25.7)	172	(42.4)	234	(57.6)	99	(24.4)	307		(75.6)
T3/T4a/T4b		1174	(74.3)	404	(34.4)	770	(65.6)	341	(29.0)	833		(71.0)
**N-Category**	*n p*-Value_(b)_	1561		1561		0.843		1561		**< 0.001**		
N0		866	(55.5)	314	(36.3)	552	(63.7)	203	(23.4)	663		(76.6)
N1a/b/c		334	(21.4)	115	(34.4)	219	(65.6)	101	(30.2)	233		(69.8)
N2a/b		361	(23.1)	136	(37.7)	225	(62.3)	129	(35.7)	232		(64.3)
**N-Category**	*n p*-Value_(a)_	1561		1561		0.958		1561		**< 0.001**		
N0		866	(55.5)	314	(36.3)	552	(63.7)	203	(23.4)	663		(76.6)
N+ (N1a/b/c, N2a/b)		695	(44.5)	251	(36.1)	444	(63.9)	230	(33.1)	465		(66.9)
**M-Category**	*n p*-Value_(a)_	1580		1580		0.288		1568		**0.028**		
M1		182	(11.5)	73	(40.1)	109	(59.9)	63	(34.8)	118		(65.2)
MX		1398	(88.5)	503	(36.0)	895	(64.0)	373	(26.9)	1014		(73.1)
**UICC Stage**	*n p*-Value_(b)_	1580		1580		0.349		1580		**< 0.001**		
I		330	(20.9)	142	(43.0)	188	(57.0)	77	(23.3)	253		(76.7)
IIA/B/C		519	(32.8)	169	(32.6)	350	(67.4)	122	(23.5)	397		(76.5)
IIIA/B/C		550	(34.8)	193	(35.1)	357	(64.9)	179	(32.5)	371		(67.5)
IVA/B		181	(11.5)	72	(39.8)	109	(60.2)	62	(34.3)	119		(65.7)
**L-Category**	*n p*-Value_(a)_	1580		1580		0.306		1580		**0.008**		
L0		1184	(74.9)	423	(35.7)	761	(64.3)	309	(26.1)	875		(73.9)
L1		396	(25.1)	153	(38.6)	243	(61.4)	131	(33.1)	265		(66.9)
**V-Category**	*n p*-Value_(a)_	1579		1579		0.312		1579		0.828		
V0		1466	(92.8)	540	(36.8)	926	(63.2)	409	(27.9)	1057		(72.1)
V1		113	(7.2)	36	(31.9)	77	(68.1)	30	(26.5)	83		(73.5)
**Pn-Category**	*n p*-Value_(a)_	722		722		0.872		722		0.605		
Pn0		677	(93.8)	231	(34.1)	446	(65.9)	184	(27.2)	493		(72.8)
Pn1		45	(6.2)	16	(35.6)	29	(64.4)	14	(31.1)	31		(68.9)
**Grading**	*n p*-Value_(a)_	1540		1540		0.837		1540		0.942		
Low grade (G1/G2)		1254	(81.4)	442	(35.2)	812	(64.8)	347	(27.7)	907		(72.3)
High grade (G3/G4)		286	(18.6)	103	(36.0)	183	(64.0)	80	(28.0)	206		(72.0)
**R-Status**	*n p*-Value_(a)_	1535		1535		0.744		1535		0.053		
R0		1494	(97.3)	544	(36.4)	950	(63.6)	410	(27.4)	1084		(72.6)
R1/R2		41	(2.7)	16	(39.0)	25	(61.0)	17	(41.5)	24		(58.5)
**Overall Survival [Months]**	*p*-Value_(c)_			1576		0.414		1576		**0.007**		
Total / Events / Censored		1576/781/795		575/281/294		1001/500/501		437/244/193		1139/537/602		
Median Survival		100.0		106.0		87.1		64.3 ± 10.0		127.2		
95% C.I.		n.c.		n.c.		n.c.		44.5 - 84.1		n.c.		
Survival after 5 years in %		56.6 +/- 1.3		n.c.		n.c.		51.4 +/- 2.4		58.6 +/- 1.5		
Survival after 10 years in %		47.5 +/- 1.4		n.c.		n.c.		40.8 +/- 2.6		50.1 +/- 1.6		
**UICC II-Subgroup: Overall Survival**	*p*-Value_(c)_							519		**0.043**		
Total / Events / Censored								121/61/60		396/157/239		
Median Survival								106.1 +/- 17		n.c.		
95% C.I.								n.c.		n.c.		
UICC stage II: survival after 5 years in %								59.3 +/- 4.5		69.0 +/- 2.3		
UICC stage II: survival after 10 years in %								44.7 +/- 5.1		57.0 +/- 2.7		
**Tumor Specific Survival [Months]**	*p*-Value_(c)_			1559		0.205		1559		**0.011**		
Total / Events / Censored		1559/526/1033		571/183/388		988/343/645		431/168/263		1128/358/770		
Median Survival		n.c.		n.c.		n.c.		n.c.		n.c.		
95% C.I.		n.c.		n.c.		n.c.		n.c.		n.c.		
Survival after 5 years in %		66.8 +/- 1.2		n.c.		n.c.		61.6 +/- 2.4		68.8 +/- 1.4		
Survival after 10 years in %		62.7 +/- 1.4		n.c.		n.c.		56.7 +/- 2.7		65.0 +/- 1.6		
**UICC II-Subgroup: Tumor Specific Survival**	*p*-Value_(c)_							519		**0.045**		
Total / Events / Censored								119/34/85		394/80/314		
Median Survival								n.c.		n.c.		
95% C.I.								n.c.		n.c.		
UICC stage II: survival after 5 years in %								73.1 +/- 4.2		81.8 +/- 2.0		
UICC stage II: survival after 10 years in %								66.0 +/- 5.2		76.9 +/- 2.4		

^(a)^ Fisher‘s exact test

^(b)^ Kendall‘s tau test

^(c)^ Log-rank test

Abbreviations: n.c. = not calculated.

### ERG and insulin receptor immunohistochemistry

1580 primary CRC samples obtained from 1580 patients were assessed for the expression of IR in endothelial cells of tumor vessels (VIR) and tumor cells (EIR). The presence of tumor vessels was confirmed by immunostaining with an anti-ERG-antibody in every case.

**Figure 1:**
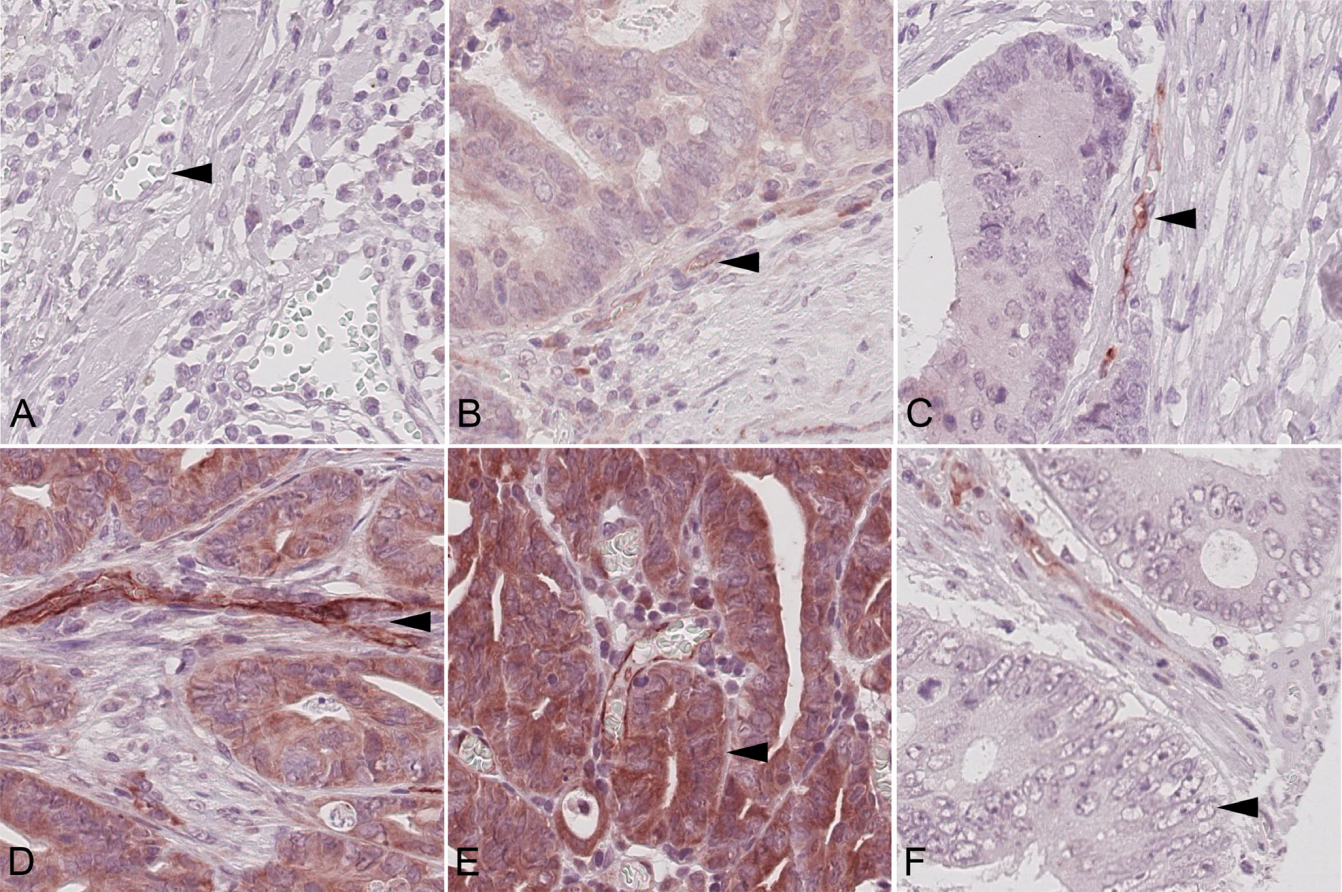
Insulin receptor immunoreactivity Colorectal carcinoma samples showing (**A**) none (VIR-0), (**B**) weak (VIR-1+), (**C**) moderate (VIR-2+), and (**D**) strong (VIR-3+) vascular insulin receptor staining (arrow heads). Examples of (**E**) epithelial insulin receptor overexpression (EIR; arrow head) and absence of EIR (**F**; arrow head). Magnification A–F: 400x.

With regard to VIR, no immunostaining was found in 181 (VIR-0; 11.5%; Figure 1A) cases. A weak expression was found in 395 (VIR-1+; 25%; Figure 1B) cases, a moderate in 879 (VIR-2+; 55.6%; Figure 1C) and a strong in 125 (VIR-3+; 7.9%; Figure 1D). We observed the IR to be particularly overexpressed in capillaries. The staining was weaker in arterioles or venules.

No VIR was observed in healthy controls of colonic mucosa (*n* = 10) or in areas of non-neoplastic colonic mucosa (*n* = 5; Supplementary Figure 1) of exemplarily generated CRC whole tissue-slides.

1140 (72.2%) CRCs showed expression of the IR in tumor cells (EIR; Figure 1E), with membranous staining being observed in 224 (19.7%) and cytoplasmic in 1118 (98.3%) CRCs. Both, membranous and cytoplasmic staining was found in 205 (18.0%) cases. In 3 cases we were unable to differentiate between membranous and cytoplasmic immunostaining.

### Insulin receptor *in situ* hybridization

**Figure 2:**
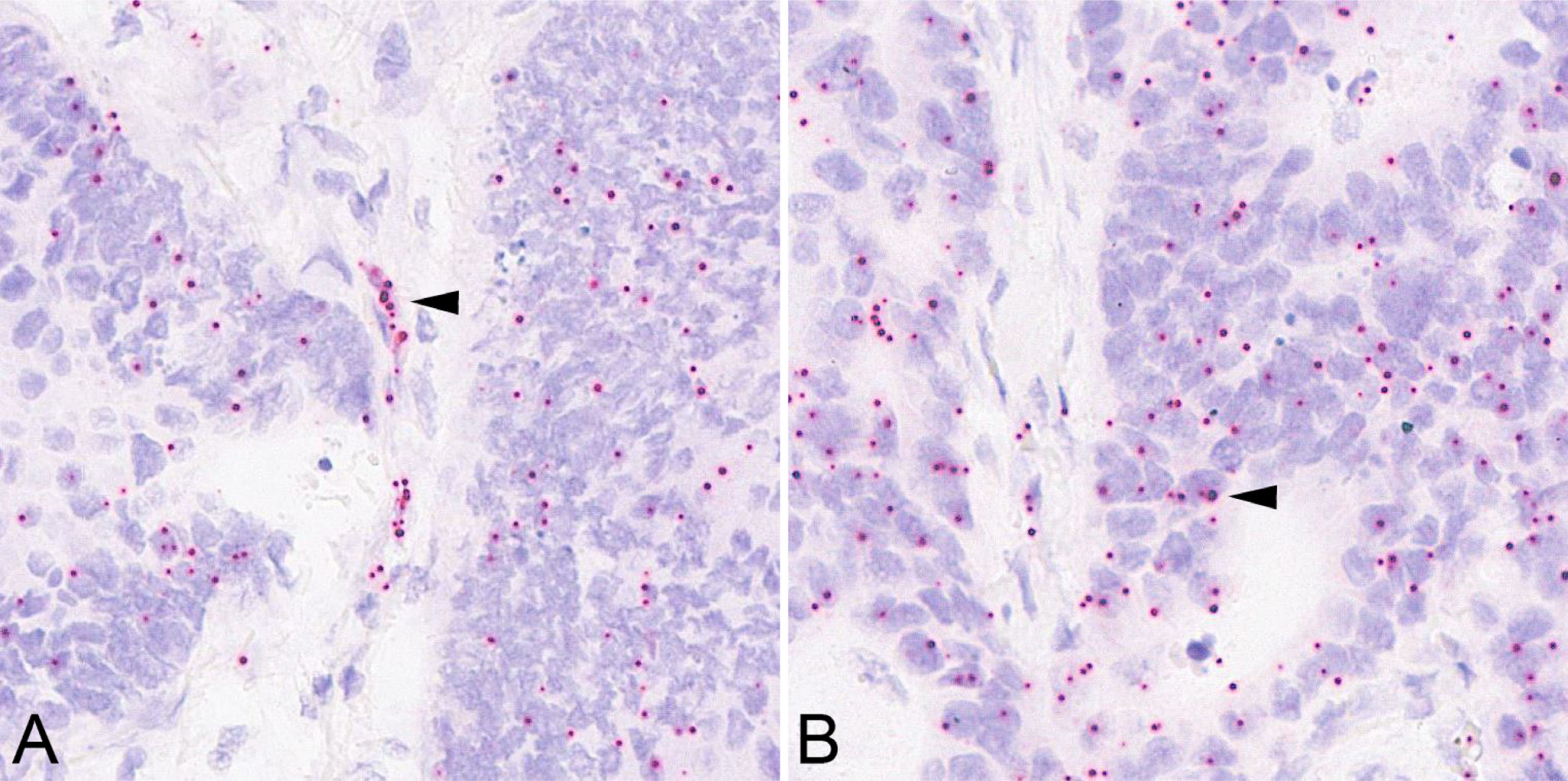
Insulin receptor isoform A *in situ* hybridization As determined by *in situ* hybridization, the vascular (**A**; arrow head) and epithelial (**B**; arrow head) overexpression of insulin receptor isoform A mRNA correlated with insulin receptor immunoreactivity. Magnification A–B: 400x.

Using *in situ* hybridization we wished to test whether the IR-A is the preferred isoform expressed in tumor cells and tumor vessels of CRCs, respectively. To this end, we generated tissue microarrays (TMA), which enclosed 125 CRCs with VIR-3+. We added randomly selected existing TMAs from our collective containing 141 cases with a VIR below 3+ for comparison. H&E staining was used to confirm successful transfer of VIR 3+ tumor tissue and whether the core cylinders enclosed tumor cells and tumor vessels. Unfortunately, the core cylinders of 15 CRCs did not contain tumor vessels and were excluded from the analysis. Finally, 110 CRCs with VIR-3+ could be analyzed. The expression of IR-A was confirmed in 108 (98.2%) cases with VIR-3+ (Figure 2A).

The VIR score, which was employed for the evaluation of IR immunohistochemistry correlated significantly with IR-A expression as visualized by *in situ* hybridization (*P* = 0.032). The higher the immunohistological VIR score, the more frequently vascular IR-A signals could be detected by *in situ* hybridization (Supplementary Table 1).

Next we analyzed tumor cells (Figure 2B) irrespective of the VIR-status. The number of IR-A mRNA signals was counted in 100 tumor cells and a ratio was calculated using the formula: number of signals divided by the number of tumor cells. The median ISH-ratio was found to be 0.36 (range 0.05–1.48).

### Correlation of insulin receptor – expression with clinico-pathological data

**Figure 3:**
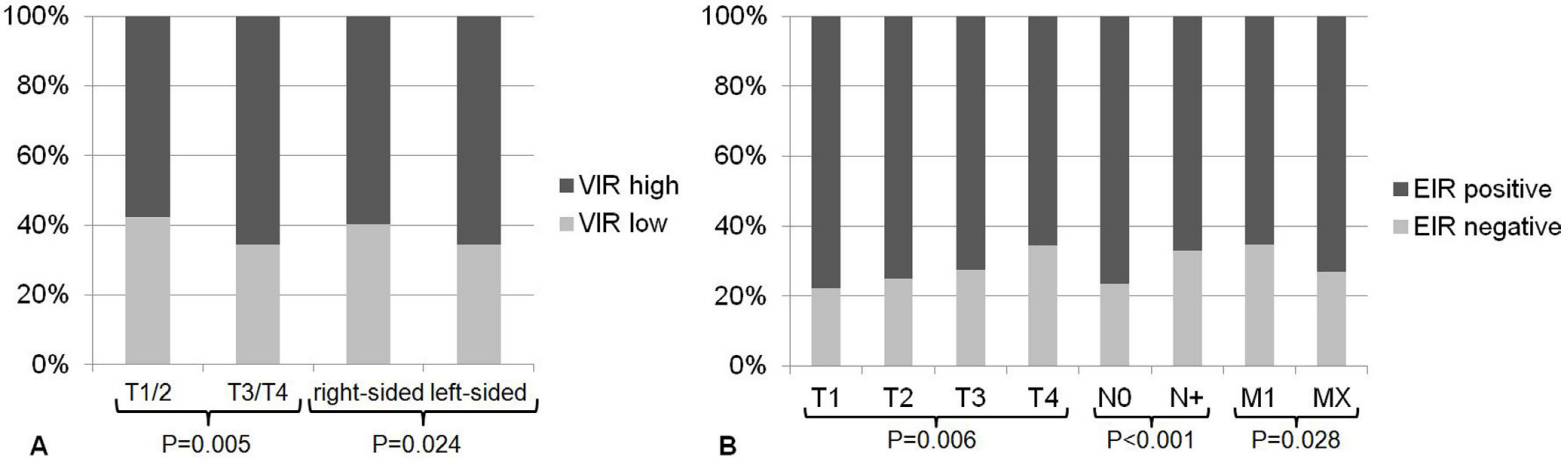
Association between insulin receptor expression and clinico-pathological parameters (**A**) VIR was significantly associated with the T-stage (*P* = 0.005), being higher in T3/4 tumors compared with T1/T2-tumors. VIR-high was significantly more frequent in left-sided CRCs (*P* = 0.024). (**B**) EIR was significantly associated with the T-stage, lymph node metastasis (N-category), distant metastasis (M-category), lymphatic invasion (L-category) and UICC stage (not shown).

For statistical analyses we dichotomized the intensity of VIR expression into a VIR-low (VIR-0; VIR1+) and VIR-high (VIR-2+; VIR-3+) group, constituting 1004 (63.5%) cases with VIR-high and 576 (36.5%) with VIR-low. VIR correlated significantly with local tumor growth, being higher in T3/4 tumors compared with T1/T2-tumors (*P* = 0.005; Table 1, Figure 3A). VIR-high was also significantly more prevalent in left-sided CRCs (*P* = 0.024; Table 1, Figure 3A), which included all CRCs located at and aborally of the left flexure. No further correlations were found between VIR and any other clinico-pathological patient characteristics (Table 1).

EIR positivity (Figure 1E) was defined as the presence of any membranous or cytoplasmic immunostaining and it was distinguished from EIR negativity, which was defined as the absence of any EIR immunostaining (Figure 1F). Statistical analysis of the EIR status as such revealed that EIR correlated significantly with local tumor growth (T-category), nodal spread (N-category), lymphatic invasion (L-category), distant metastasis (M-category) and UICC stage (Table 1, Figure 3B). An inverse association existed between the T-category and EIR: 77.9% of the T1-tumors showed EIR expression compared with 65.4% in T4-tumors (*P* = 0.006). Similarly, the prevalence of EIR differed with 76.7% in UICC stage I tumors from 65.7% in UICC stage IV tumors. Lymph node metastasis and lymphatic invasion were significantly associated with EIR negativity. CRC patients without lymph node metastasis displayed EIR in 76.6%, whereas primary tumors of CRC patients with lymph node metastasis only showed EIR in 66.9% (*P* < 0.001). Primary CRC samples without lymphatic invasion expressed the IR in 73.9%, whereas only 66.9% exhibited EIR when lymphatic invasion was present.

Primary tumors of patients with metastatic CRC at the time of primary surgery displayed EIR less frequently (65.2%) when compared with patients without distant metastases at the time of primary surgery (73.1%; *P* = 0.028).

### Survival analysis

The entire CRC cohort showed an overall survival (OS) of 56.6 ± 1.3% after 5 years and of 47.5 ± 1.4% after 10 years. The tumor specific survival (TSS) was 66.8 ± 1.2% after 5 years and 62.7 ± 1.4% after 10 years.

**Figure 4:**
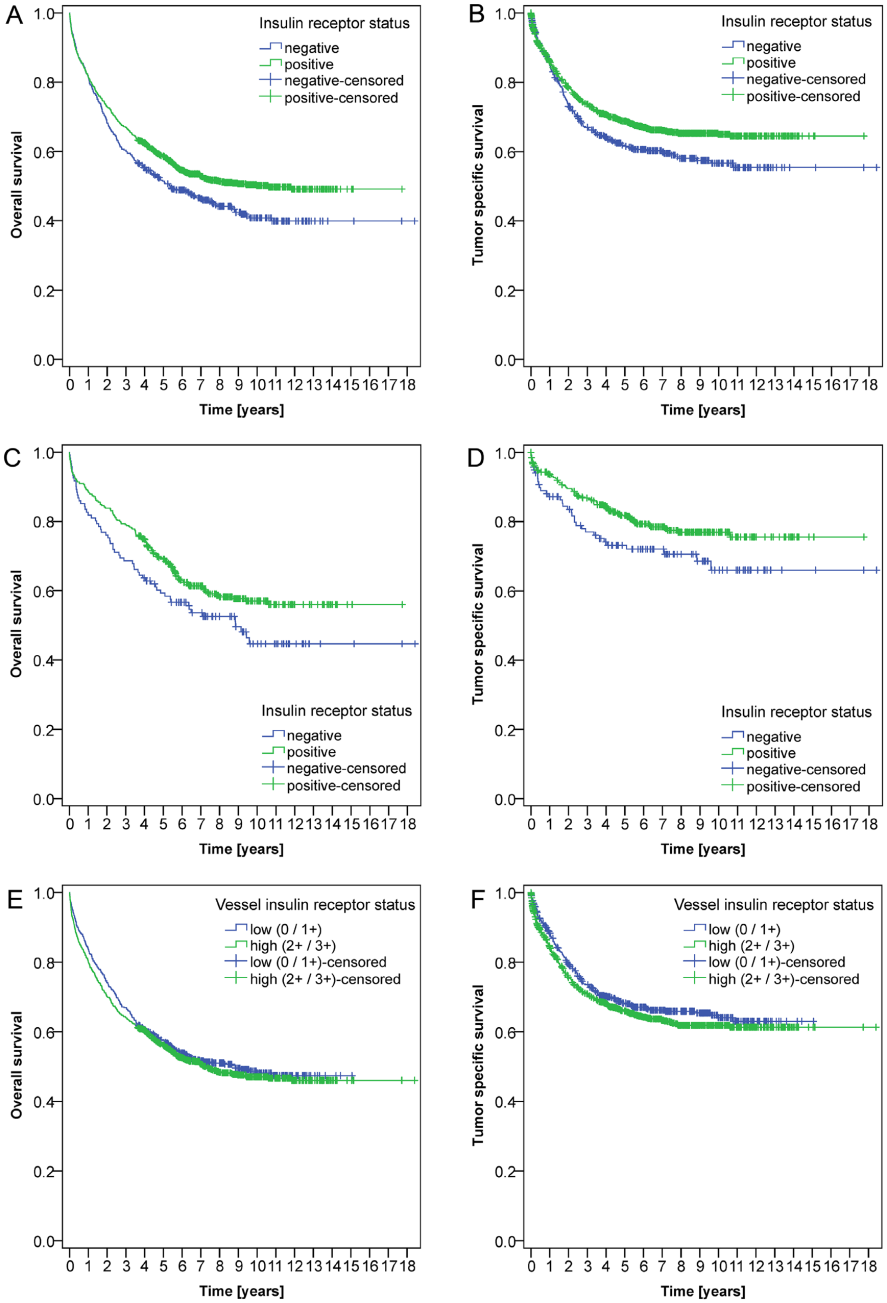
Kaplan-Meier curves Kaplan-Meier curves depict significant correlations between the expression of the insulin receptor in tumor cells (EIR) and overall (**A**; *P* = 0.007) and tumor specific survival (**B**; *P* = 0.011) in the entire cohort. Furthermore, patients without epithelial IR expression (EIR-negative) showed a worse prognosis in UICC stage II CRC with respect to overall (**C**; *P* = 0.043) and tumor specific survival (**D**; *P* = 0.045). Vascular IR expression (VIR) was not associated with overall (**E**, *P* = 0.414) or tumor specific survival (**F**, *P* = 0.205).

The expression of IR in tumor cells (EIR) correlated significantly with OS (*p* = 0.007) (Figure 4A) and TSS (*p* = 0.011) (Figure 4B): When performing a subgroup analysis of the clinically relevant UICC stage II group, a significant difference with regard to OS (*p* = 0.043) (Figure 4C) and TSS was observed (*p* = 0.045) (Figure 4D).

No significant correlation could be found between VIR and either OS (*P* = 0.414) (Figure 4E) or TSS (*P* = 0.205) (Figure 4F).

### Correlation of vascular and epithelial insulin receptor overexpression

A comparative analysis yielded a significant and positive correlation between VIR and EIR (*P* < 0.001). A simultaneous expression of IR by tumor cells was found in 83.2% of the VIR-high group. To the contrary, only 53.0% of the VIR-low group showed IR-expression by tumor cells. When looking at the individual VIR scores a gradual increase of tumor cells expressing the IR could be observed along with increasing VIR scores: CRC samples with no (VIR 0), weak (VIR 1+), moderate (VIR 2+) or strong (VIR 3+) endothelial immunostaining presented tumor cell staining in 32.0%, 62.5%, 82.0% or 91.2% of the cases, respectively (*P* < 0.001).

### DNA mismatch repair protein expression

DNA mismatch repair protein (MMR) expression in patients with UICC stage II CRC was studied in 517 out of 519 patients. Two patient samples were excluded due to technical artifacts. 80.5% (416) of all patients were MMR proficient. An MMR deficiency (dMMR) was observed in 19.5% (101) of all patients with UICC stage II CRC. Patients with dMMR showed a tendency for a better outcome, but missed significance with respect to overall survival (*P* = 0.239) and tumor specific survival (*P* = 0.293).

The dMMR status correlated significantly with the anatomical location of the CRC: it was significantly more prevalent in right-sided (32.2%) compared with left-sided CRCs (8.8%; *P* < 0.001).

Insulin receptor expression in tumor cells (EIR) tended to be associated with dMMR in UICC stage II CRC: EIR negative patients tended to exhibit dMMR less frequently (16.8%) than patients with EIR positive CRCs (83.2%; *P* = 0.089).

A similar tendency was observed with respect to vascular IR expression (VIR) in UICC stage II CRC: dMMR tended to be less frequent in patients of the VIR-low group (25.7%) than in patients of the VIR-high group (74.3%; *P* = 0.124).

## DISCUSSION

Our comprehensive analysis of the expression of the insulin receptor in a large CRC cohort encompassing 1580 cases provides evidence that the IR is differentially expressed in both, tumor cells (EIR) and endothelial cells (VIR) of tumor vessels. *In situ* hybridization confirmed the preferred expression of the isoform A. We correlated IR expression with various clinico-pathological patient characteristics and survival, and found a significant association between VIR expression and local tumor growth as well as anatomical site. VIR was significantly more commonly observed in left-sided CRCs. With regard to tumor cells, EIR expression correlated significantly with local tumor growth, lymph vessel invasion, nodal spread, distant metastases, tumor stage and even patient survival. Collectively, our data support the contention that the expression of the IR might be of tumor biological significance in CRC in a cell type specific manner.

Using immunohistochemistry, VIR expression has recently been described in invasive bladder cancer by Roudnicky et al. [11] as a predictor of poor overall and progression-free survival (*n* = 63). They further studied five representative samples via RT-PCR and found IR-A to be upregulated in VIR.

Although we visualized vascular IR-A expression in CRC by *in situ* hybridization, our detection method of vascular IR-A differed from their study, which, to some extent, limits the comparability. However, our studies are comparable with respect to the immunohistological evaluation and correlation with clinico-pathological parameters and survival analysis, as we employed an almost identical scoring system (no expression (0), weak expression (1+), strong expression (2+)) and the same dichotomization (VIR-low with no or weak vascular expression versus VIR-high with high vascular IR expression) and also used TMAs. We did not find a significant correlation between VIR and either OS or TSS in CRC, despite having analyzed a substantially larger collective. We therefore speculate that the influence of VIR on survival might depend on the tumor type.

The correlation observed between VIR and the T-stage in CRC could be explained by the pathophysiological findings of Roudnicky et al., who described endothelial IR expression to be up-regulated upon hypoxia *in vitro* [11]. They hypothesized that hypoxia-associated VIR upregulation might be controlled by HIF1α in endothelial cells. Endothelial IR upregulation was accompanied by an up-regulation of the hypoxia-associated marker GLUT1 *in vitro* in human umbilical vein endothelial cells and histologically in bladder cancer endothelial cells [11]. As cancer sites with higher T-stages have an increased risk of developing hypoxic states, we suggest that this could explain the high frequency of VIR in our T3/T4 stage CRCs. In line with the concept of VIR’s upregulation especially under hypoxic conditions, we think of VIR as a new contributing force to neovascularization in CRC. Liu et al. [12] demonstrated that insulin induces endothelial cell migration and tube formation *in vitro*, which was mediated by the IR and lead to an elevation of VEGF levels in the endothelium. Interestingly, upon VEGF receptor inhibition, insulin still induced endothelial migration via the IR. Roudnicky et al. [11] demonstrated that IGF-II was a strong stimulator of endothelial cell migration *in vitro* and that this effect could be abolished completely by IR inhibition. Then an endothelial IR knockout mouse model revealed, that angiogenesis was not affected by endothelial IR deletion *in vivo*. Therefore a redundancy of angiogenetic pathways can be assumed, which step in when vascular IR is deleted. Nevertheless based upon our new findings of a high frequency of VIR in T3/T4 CRCs and the concordant experimental data of the studies mentioned above, VIR seems to play a role in neovascularization. This might explain the worse disease-free survival and increased local recurrence of CRC in patients with metabolic syndrome [13]. Elevated insulin levels might contribute to neovascularization via VIR. Further studies are needed to elucidate this pathophysiological mechanism.

EIR was present in the majority of our cases. We noticed that EIR was found almost exclusively in the cytoplasm of tumor cells, whereas a (simultaneous) membranous IR expression was less prevalent. These findings could be explained with *in vitro* experiments by Morcavallo et al., who demonstrated that upon insulin or IGF-II stimulation the phosphorylated and thus activated IR-A internalizes from the cell surface [14]. Their experimental results suggested that the mechanism of IR-A endocytosis is associated with a sustained receptor phosphorylation and therefore prolonged activation [14].

Our findings about EIR in CRC go beyond the results of Abbruzzese et al. [15], who examined EIR immunohistochemically in a smaller (117 patients) CRC collective: They described the phosphorylated IR to be expressed in 41.9% of CRC patients and documented a significantly higher frequency of EIR in low-grade CRC. EIR positive CRC patients had an improved disease-free survival in their collective. We could broaden the picture of EIR in CRC by studying a larger patient collective: We showed that distant metastasis, lymphatic invasion, lymph node metastasis, TSS and OS were also significantly associated with EIR in CRC.

Abbruzzese et al. suggested that EIR could be regarded as a “normal” initial state of CRC development, which has to be elucidated by future prospective studies.

We suggest that the tumor (sub-) type also determines the correlation between EIR and clinico-pathological parameters as well as patient survival. This notion is supported by the comparison with other immunohistological studies about EIR in different cancer entities, which reveals that the associations with IR expression differ between tumor types:

EIR has been associated with worse survival rates in non small cell lung cancer [7]. In breast cancer, EIR had been linked with either worse or improved survival rates, depending on the study at hand [8, 9]. It has to be noted that breast cancer represents a heterogeneous disease, which becomes evident when studying IGF-I-R expression for instance: A meta-analysis about IGF-I-R expression in breast cancer had revealed that IGF-I-R overexpression was a favorable prognostic variable in unselected breast cancers, but lead to a reduced survival in triple-negative breast cancers [16]. Takahashi et al. [10] studied IR expression in renal cell cancer and demonstrated EIR to be higher in lower tumor stages and to be associated with a better prognosis.

The decision for chemotherapy in patients with UICC stage II CRC is subject to the individual evaluation of risk factors. Microsatellite instability (MSI) has been found to be associated with a significantly better recurrence-free and OS in UICC stage II CRC patients [17] and influences contemporary therapy algorithms. We assessed the influence of the MMR status in the subgroup of UICC stage II patients in our patient cohort and found a tendency for an improved outcome in dMMR patients. The analysis did not reach significance, which might be explained by the circumstance that a fraction of the patient group had received chemotherapy, which might have shifted the overall outcome. It has to be noted, that due to the large patient numbers in our study and reasons of practicability, we chose not to test the tumor samples genetically and solely relied on assessing the MMR status immunohistochemically. Immunohistochemistry usually suffices for the evaluation of the MMR status, but bears the possibility of missing a minority of cases with MSI. Nevertheless, we observed a significant association of the dMMR status and right-sidedness which has been repeatedly published by other groups [18], thereby indirectly supporting the validity of our data. If the MMR status influences survival significantly, is still the subject of ongoing discussions: Gkekas et al. [19] recently published a meta-analysis which found no significant associations between survival and the MSI status in UICC stage II CRC patients, therefore questioning its role in therapeutic algorithms.

A negative EIR status in CRC seems to identify patients at risk and might help with respect to clinical decision making processes:

23.5% of our CRC patients with UICC stage II were negative for EIR. A separate survival analysis revealed that OS and TSS were significantly worse in EIR negative UICC stage II CRC patients despite an adequate therapy. We therefore identified EIR negativity in UICC stage II patients as a new potential risk factor for this patient subgroup.

We propose further prospective studies to investigate, if EIR negative UICC stage II CRC patients could profit from adjuvant chemotherapy as a risk group.

In the light of the high frequency of IR expression in our CRC cohort, the natural formation of IR / IGF-I-R-hybrids can be assumed. The employed monoclonal IR antibody might not bind IR / IGF-I-R-hybrids, as it is highly specific for IR. It would be of interest to investigate the presence of IR / IGF-I-R-hybrids in future studies.

In conclusion, we demonstrated VIR to be a frequent phenomenon in CRC and to be significantly associated with the tumor size (T-category) and localization. VIR seems to be a new cornerstone of angiogenesis in CRC, which has to be further elucidated by future studies.

EIR was common in CRC and its frequency decreased with increasing tumor stages. EIR immunonegativity was associated with a more aggressive CRC phenotype in our cohort. The decision for or against chemotherapy in UICC stage II CRC patients is based on the evaluation of risk factors. We identified EIR negativity in UICC stage II CRC as a relevant risk factor, which was significantly associated with worse survival in this subgroup. This new risk factor’s potential to influence therapeutic decision making in UICC stage II CRC should be evaluated in future prospective studies. Due to their high frequency in CRC, VIR and EIR should also be exploited for targeted therapeutic approaches.

## MATERIALS AND METHODS

### Ethics statement

All tissue samples were obtained as part of a diagnostic or therapeutic surgery carried out after the patients had given written informed consent. The study was carried out in accordance to the Declaration of Helsinki. All patient data were pseudonymized prior to study inclusion. Ethical approval for the study was provided by the ethical review board of the Medical Faculty, Christian-Albrechts-University Kiel, Germany (reference number D 438/17).

### Study population and histology

From the archive of the Department of Pathology of the Christian-Albrechts-University in Kiel, all patients who had undergone oncologic resection of primary CRC between 1995 and 2011 were retrieved. Tissue specimens had been fixed in neutral buffered formalin and embedded in paraffin. Paraffin sections were stained with hematoxylin and eosin (H&E). The histological classification was carried out according to the World Health Organization-criteria. The tumor-node-metastasis stage was determined according to the *union internationale contre le cancer* (UICC; 7th edition) [20] and was based on histological confirmation by board certified pathologists.

Patients were excluded (1) if tissue samples did not contain tumor cells and tumor vessels and (2) if they suffered from syn- or metachronous colon cancer.

Biopsies of colonic mucosa stemming from diagnostic colonoscopies of 10 different individuals which had been histopathologically classified as being normal served as healthy controls.

### Tissue microarray construction and immunohistochemistry

Formalin-fixed and paraffin-embedded tissue samples were used to generate tissue microarrays (TMA) as described in detail previously [21]. Three different representative areas of the CRCs were chosen within the respective H&E-stained slides and three cores were punched for every CRC sample.

10 healthy colonic biopsies and 5 CRC whole tissue-slides containing areas of non-neoplastic mucosa were stained for IR expression as well.

For immunohistochemical staining, paraffin sections were deparaffinized and boiled in EDTA buffer (pH 9.0) for 1 min at 125°C. After washing with Tris-buffered saline (TBS), slides were blocked with hydrogen peroxide block (Thermo Fisher Scientific) for 15 min followed by washing with TBS and treatment with Ultra V Block (Thermo Fisher Scientific) for 5 min. Incubation with the primary antibodies was done overnight at 4°C after incubation for 30 min at room temperature. The following antibodies and dilutions were used: anti-ERG antibody (Zytomed Systems GmbH, Berlin, Germany) 1:50, anti-insulin receptor β-antibody (rabbit monoclonal; clone 4B8; Cell Signaling Technologies, Danvers, USA) 1:50. Immunoreactions were visualized with the ImmPRESS reagent peroxidase universal anti-mouse/rabbit Ig – MP-7500 (Vector Laboratories, Burlingame, CA, USA) and ImmPact NovaRed peroxidase substrate SK-4805 Kit (Vector Laboratories, Burlingame, CA, USA). The specimens were counterstained with hematoxylin. Omission of the primary antibody served as negative controls. Endometrium samples were used as positive controls.

TMAs containing UICC stage II CRC samples were stained for the DNA mismatch repair (MMR) proteins MLH1, PMS2, MSH2 and MSH6. The TMAs contained three cores per patient. The immunostainings were performed with antibodies directed against MLH1 (mouse monoclonal; BD Pharmingen, San Diego, USA) 1:20, PMS2 (mouse monoclonal; Cell Marque, Rocklin, CA, USA) 1:20, MSH2 (mouse monoclonal; Calbiochem, San Diego, USA) 1:100 and MSH6 (mouse monoclonal, BD Transduction Laboratories, BD Biosciences, San Jose, CA, USA) 1:50, on the autostainer Bond^™^ Max System (Leica-Menarini, Berlin, Germany). Antigen retrieval was carried out using the ER2 antigen retrieval solution for 20 min at pH 9.0 (Leica-Menarini) for MLH1, PMS2 and MSH2. For MSH6, antigen retrieval was performed using the ER1 antigen retrieval solution for 20 min at pH 6.0 (Leica-Menarini) and the DAB Enhancer (Leica-Menarini) was employed to enhance the immunostaining.

### Evaluation of ERG and insulin receptor immunostaining

All CRC samples were evaluated for the presence of the vessel marker ERG. A sample was to be excluded from the study, if the ERG-staining should not confirm the presence of tumor vessels. A tumor vessel was defined as a vessel surrounded by epithelial cancer cells within a tumor site.

Insulin receptor immunostaining of epithelial tumor cells (EIR) and endothelial cells of tumor vessels (VIR) was assessed separately.

The intensity of VIR was classified according to the following scoring system. The maximum intensity of immunostaining observed in a given tumor was categorized as negative (0), weak (1+), moderate (2+) and strong (3+). The vessel with the most intense staining determined the overall sample’s category. Pre-selected reference-slides representing the four different categories served as a control throughout the evaluation process and reduced the risk of intraobserver variance (Figure 1). The vascular immunostaining categories were subsequently dichotomised into the negative / low VIR group enclosing cases with none (0) or weak (1+) immunostaining of vascular endothelial cells (1) and the high VIR group with cases harboring moderate (2+) or strong (3+) immunostaining of vascular endothelial cells.

The VIR status was also assessed in 10 healthy colonic biopsies and within the non-neoplastic mucosa of 5 CRC whole tissue-slides.

EIR immunostaining was classified as either being positive, if any membranous or cytoplasmic immunostaining was evident, or negative, if no immunostaining was present (Figure 1). Immunostaining of different cellular compartments (membranous or cytoplasmic) of EIR was noted and categorized as positive, if any staining was noted, and negative, lack of any immunostaining.

### Evaluation of DNA mismatch repair protein immunostaining

DNA mismatch repair (MMR) proteins MLH1, PMS2, MSH2 and MSH6 were evaluated according to the presence or absence of nuclear staining, employing the algorithm suggested by Remo et al. [22]. We hereby distinguished MMR proficient from MMR deficient (dMMR) CRCs. CRCs were excluded, if the staining for MMR proteins could not be conclusively evaluated, e.g. in the case of a weak staining result with absent positive internal controls, or due to technical artefacts such as fragmented sample cores.

### Insulin receptor isoform A *in situ* hybridization

*In situ* hybridization was done by Creative Bioarray (Shirley, NY, USA) and Advanced Cell Diagnostics (Newark, CA, USA). The probe consisted of two parts (RNAscope^®^ ISH technology) which bound to the defining region 2612–2651 of the IR-A mRNA (NM_001079817.2).

Successful *in situ* hybridization (ISH) was assessed as follows: vascular IR-A mRNA expression was classified as either being positive, if vascular mRNA expression could be detected, or negative, if vascular insulin receptor isoform A mRNA was not present. Subsequently, epithelial IR-A mRNA ISH-signals were counted per 100 epithelial CRC cells and a ratio was calculated (epithelial ISH signals / 100 cancer cells = ISH-ratio).

### Statistical analyses

Statistical analyses were done using SPSS version 24.0 (IBM Corp., Armonk, NY, USA). Fisher’s exact test was used to test the correlation between the VIR status or the EIR status and non-ordinal clinico-pathological patient characteristics or the dMMR status respectively. Kendall’s tau-test was used for variables of ordinal scale such as T-category, N-category, UICC-stage, tumor grading and for the comparison of vascular IR expression as observed in immunohistochemistry versus *in situ* hybridization. The Kaplan-Meier method was used to determine the median survival with 95% confidence intervals. The log rank test was employed for testing differences between median survivals. A *P*-value of ≤ 0.05 was regarded as being significant.

## SUPPLEMENTARY MATERIALS FIGURE AND TABLE



## Abbreviations

CRC: colorectal cancer
dMMR: DNA mismatch repair protein deficiency
EIR: insulin receptor expression in epithelial tumor cells
IGF-II: insulin like growth factor II
IGF-I-R: insulin like growth factor receptor I
IR: insulin receptor
IR-A: insulin receptor isoform A
IR-B: insulin receptor isoform B
ISH: *in situ* hybridization
ISH-ratio: epithelial ISH signals / 100 cancer cells
MMR: DNA mismatch repair proteins
MSI: microsatellite instability
OS: overall survival
RT-PCR: reverse transcription polymerase chain reaction
TMA: tissue microarray
TSS: tumor specific survival
UICC: union internationale contre le cancer
VIR: insulin receptor expression in tumor-vasculature
