# Supplementary material for: Epithelial insulin receptor expression–prognostic relevance in colorectal cancer
Source: Oncotarget. 2018 Dec 25;9(101):37497–508. doi: 10.18632/oncotarget.26490 (PMC6331016; doi:10.18632/oncotarget.26490)
Supplement: Supplementary file 1 [file oncotarget-09-37497-s001.pdf]

Epithelial insulin receptor expression–prognostic relevance in colorectal cancer

SUPPLEMENTARY MATERIALS

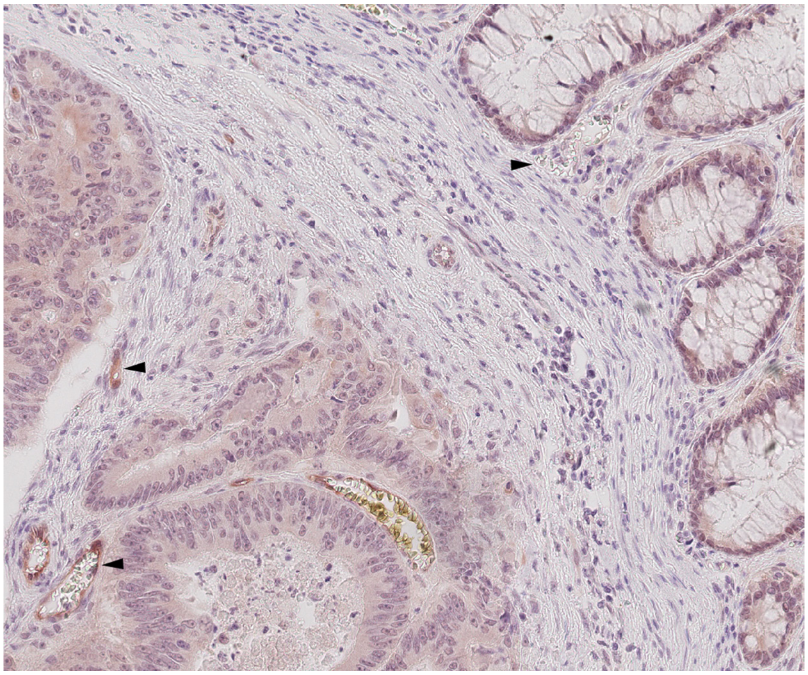

Supplementary Figure 1: Example of vascular insulin receptor expression within the tumor site (arrow heads, lower left corner) and adjacent normal mucosa without vascular insulin receptor expression (arrow head, upper right corner) in a CRC whole tissue-slide.

Supplementary Table 1: Vascular insulin receptor expression as assessed by immunohistochemistry versus *in situ* hybridization

VIR score vascular insulin receptor expression (immunohistochemistry)	Vascular insulin receptor isoform A expression (*in situ* hybridization)	
	negative	positive
	n (%)	*n* (%)
0	2 (28.6)	5 (71.4)
1	1 (7.7)	12 (92.3)
2	2 (2.7)	73 (97.3)
3	2 (1.8)	108 (98.2)

P = 0.032 (Kendall's tau-b)
